# Association between Fas/FasL gene polymorphism and musculoskeletal degenerative diseases: a meta-analysis

**DOI:** 10.1186/s12891-018-2057-z

**Published:** 2018-05-07

**Authors:** Donghua Huang, Jinrong Xiao, Xiangyu Deng, Kaige Ma, Hang Liang, Deyao Shi, Fashuai Wu, Zengwu Shao

**Affiliations:** 1 Department of Orthopaedics, Union Hospital, Tongji Medical College, Huazhong University of Science and Technology, 1277 JieFang Avenue, Wuhan, 430022 China; 2 Department of Epidemiology and Biostatistics, Ministry of Education Key Laboratory of Environment and Health, School of Public Health, Tongji Medical College, Huazhong University of Science and Technology, Wuhan, 430030 Hubei China

**Keywords:** Fas/FasL polymorphism, Musculoskeletal degenerative diseases, Intervertebral disc degeneration, Osteoarthritis, Rheumatoid arthritis

## Abstract

**Background:**

It was reported that Fas (rs1800682, rs2234767) and FasL (rs5030772, rs763110) gene polymorphism might be related to the risk of musculoskeletal degenerative diseases (MSDD), such as osteoarthritis (OA), intervertebral disc degeneration (IVDD) and rheumatoid arthritis (RA). However, data from different studies was inconsistent. Here we aim to elaborately summarize and explore the association between the Fas (rs1800682, rs2234767) and FasL (rs5030772, rs763110) and MSDD.

**Methods:**

Literatures were selected from PubMed, Web of Science, Embase, Scopus and Medline in English and VIP, SinoMed, Wanfang and the China National Knowledge Infrastructure (CNKI) in Chinese up to August 21, 2017. All the researches included are case-control studies about human. We calculated the pooled odds ratios (ORs) with 95% confidence intervals (95% CI) to evaluate the strengths of the associations of Fas (rs1800682, rs2234767) and FasL (rs5030772, rs763110) polymorphisms with MSDD risk.

**Results:**

Eleven eligible studies for rs1800682 with 1930 cases and 1720 controls, 6 eligible studies for rs2234767 with 1794 cases and 1909 controls, 3 eligible studies for rs5030772 with 367 cases and 313 controls and 8 eligible studies for rs763110 with 2010 cases and 2105 controls were included in this analysis. The results showed that the G allele of Fas (rs1800682) is associated with an increased risk of IVDD in homozygote and recessive models. The G allele of Fas (rs2234767) is linked to a decreased risk of RA but an enhanced risk of OA in allele and recessive models. In addition, the T allele of FasL (rs763110) is correlated with a reduced risk of IVDD in all of models. However, no relationship was found between FasL (rs5030772) and these three types of MSDD in any models.

**Conclusions:**

Fas (rs1800682) and FasL (rs763110) polymorphism were associated with the risk of IVDD and Fas (rs2234767) was correlated to the susceptibility of OA and RA. Fas (rs1800682) and Fas (rs2234767) are more likely to be associated with MSDD for Chinese people. FasL (rs763110) is related to the progression of MSDD for both Caucasoid and Chinese race groups. But FasL (rs5030772) might not be associated with any types of MSDD or any race groups statistically.

**Electronic supplementary material:**

The online version of this article (10.1186/s12891-018-2057-z) contains supplementary material, which is available to authorized users.

## Background

Degenerative disease is the consequence of a successive process resulted from degenerative cell changes, influencing tissues or organs, which will gradually deteriorate over time. The most common degenerative diseases in musculoskeletal systems include intervertebral disc degeneration (IVDD), osteoarthritis (OA) and rheumatoid arthritis (RA). IVDD, which results from ageing, small injuries and natural daily compression on intervertebral disc (IVD), has been regarded as one of the main causes to low back pain and motor deficiency. OA, a common type of joint disease, is owing to the destruction of joint cartilage and subchondral bone and is traditionally considered to be associated with articular cartilage degeneration [1]. RA, a long term autoimmune dysfunction that primarily affects joints, is also considered to be a degenerative rheumatoid and arthritis [2, 3]. All of the three diseases are of high prevalence in the society and exert huge burdens to the global medical care [4]. And they were all have been found to be related to gene alternations or heredity by recent studies [5–8].

Apoptosis represents a physiological procedure in order to remove harmful, damaged, or unwanted cells [9]. Fas is a cell-surface receptor referring to apoptotic signaling in various cell types and interacts with the natural ligand Fas ligand (FasL) to start the death signal cascade, which can contribute to apoptotic cell death [10, 11]. Fas/FasL genetic polymorphisms have been reported to be related to the development or progression of several common diseases such as cancer, systemic lupus erythematosus [12–14]. Fas (− 670 G > A rs1800682, − 1377 G > A rs2234767) and FasL (IVS2nt-124 A > G rs5030772, − 844 T > C rs763110) are the most commonly studied sites in Fas/FasL gene recently.

Although the exact etiology of OA is still unclear, current researches have explored an association between chondrocyte apoptosis and the progression of OA [15, 16]. RA, which is characterized by synovial cells proliferation and T lymphocyte collection inside the synovial tissue, is partly due to the inhibition of T cell death by which Fas/FasL participated in [17, 18]. One of the main processes in the initiation and development of IVDD is the decrease in disc cells, leading to decline in ability of synthesizing and repairing extracellular matrix [19]. Recent studies have observed a significantly higher expression levels of Fas and FasL in disc cells of the herniated lumbar disc tissues, which may result in a rapid apoptosis of resident disc cells [20, 21]. From the evidences above, we hypothesize that there may be an association of Fas and FasL gene polymorphisms with musculoskeletal degenerative diseases (MSDD). A few previous researches have reported that Fas and FasL variations were associated with these MSDD risks but came to a contradictory published results [19, 22–32]. However, no meta-analysis has investigated the association between IVDD or OA and Fas/FasL polymorphism up to now. Two meta-analyses, Zhu et al. (published in 2016) [31] and Lee et al. [33] have analyzed the association between RA and Fas/FasL recently. For Zhu et al.(published in 2016) [31] it only included and analyzed Chinese patients for Fas (rs2234767) site. For Lee et al. [33], it only analyzed Fas polymorphism and there are some mistakes in data extractions for some studies included, such as, Huang et al. [26], Lee et al. [28] and Coakley et al. [25]

So we performed a comprehensive meta-analysis containing three MSDD (OA, RA and IVDD) and enrolling all races of populations besides Chinese. Also we corrected the mistakes of the previous meta-analysis, Lee et al. [33] and added a new study, Zhu et al. (published in 2016) [31] when analyzing. This meta-analysis is designed to explore the association of MSDD (OA, RA and IVDD) with Fas/FasL polymorphism, which could assist to forecast the susceptibility of MSDD for specific individuals or conduct the clinical treatment for ‘high-risk’ individuals.

## Methods

### Strategy for literature search

To identify all literatures that studied the association of Fas and FasL genes polymorphisms with MSDD, we searched nine electronic databases including PubMed, Web of Science (WOS), Embase, Scopus and Medline in English and VIP, SinoMed, Wanfang and the China National Knowledge Infrastructure (CNKI) in Chinese. The search period for all these nine databases was up to August 21, 2017. The search strategy to explore all potential studies involved the use of the following terms: “Intervertebral Disk Degeneration” or “IDD” or “Disc Degeneration” or “disc herniation” or “low back pain” or “IVDD”, “Osteoarthritides” or “Osteoarthrosis” or “Arthritis, Degenerative” or “Degenerative Arthritis” or “Osteoarthrosis Deformans”, “Rheumatoid Arthritis”, “CD95 antigen, human” or “Fas” or “tumor necrosis factor receptor superfamily, member 6 protein, human” or “CD95L” or “Fas Ligand” or “FasL Protein” or “tumor necrosis factor ligand superfamily member 6” or “CD178 Antigens” or “CD95 Antigen Ligand” or “TNFRSF6 protein, human” or “Fas1 protein, human” or “rs1800682” or “rs2234767” or “rs5030772” or “rs763110”, “polymorphism” and “SNP”.

### Inclusion and exclusion criteria

To be included in this meta-analysis, studies should satisfy the following inclusion criteria: (1) evaluated the association of Fas and FasL genes polymorphisms with IVDD, OA and RA; (2) case-control studies; (3) offered sufficient data to calculate an odds ratio (OR) with 95% confidence interval (CI). What’s more, the following exclusion disciplines were also applied: (1) non–case-control studies; (2) repeated publications; (3) the study only concerned with a case group; (4) comment or review; and (5) not relevant to MSDD. Two investigators (Xiao and Huang) independently evaluated the articles in accord with the inclusion and exclusion criteria. Any inconsistency was solved by discussion. If these 2 authors could still not reach the uniformity, senior authors (Ma and Deng) were asked to resolve the disputes.

### Data extraction

For each study, the following characteristics were collected: (1) name of the first author; (2) year of publication; (3) country of enrollment; (4) ethnicity, age range and gender of the study population; (5) diagnosis and diagnostic criteria for MSDD cases; (6) genotyping methods; (7) source of controls; (8) matching criteria. (9) number of subjects under MSDD cases and controls; and (10) the HWE among the controls. Data were extracted cautiously from all eligible articles independently by 2 authors (Xiao and Huang). For conflict resolution, the accordance was realized by discussion.

### Methodological quality assessment

The qualities of all the included studies were assessed by two investigators (Xiao and Huang) separately using the Clark scores system, which includes 10 items [34]. Scores under 5 represent low quality; while 5–7 scores denote moderate quality and 8–10 scores indicate high quality [34].

### Statistical analysis

The PRISMA checklists and their guidelines were carefully followed in the whole process of this study [35]. The HWE in control groups for all the studies were calculated by χ^2^ test before statistical analysis and *P* <  0.05 was thought to indicate significant disequilibrium. We examined Fas (rs1800682, rs2234767) and FasL (rs5030772, rs763110) genotypes using the allele (G vs. A, C vs. T) model, homozygote (GG vs. AA, CC vs. TT) model, heterozygote (GA vs. AA, CT vs. TT) model, dominant (GG + GA vs. AA, CC + CT vs. TT) model, recessive (GG vs. GA + AA, CC vs. CT + TT) model. The strength of the association between Fas (rs1800682, rs2234767) and FasL (rs5030772, rs763110) polymorphism and MSDD was assessed by the pooled ORs and 95% CI. Subgroup analyses were conducted to find whether diagnosis of MSDD or race groups was also related to the value of the pooled ORs and 95% CI. The statistical heterogeneity was verified by *I*^*2*^ statistics. Fixed-effects model was applied to estimate the ORs and 95% CI when heterogeneity was low (*I*^*2*^ < 50%); instead, the random-effects was used when heterogeneity was high (*I*^*2*^ > 50%) [36]. Sensitivity analyses were carried out by removing one study each time to test the stability of the results. Publication bias was evaluated by the Begg’s test [37] and the Egger’s test [38] (*P* <  0.05 was considered to be statistically significant). All statistical analyses were managed using STATA 14 (Stata, College Station, TX). All *P*-values were two-sided.

## Results

### Characteristics of the studies

**Fig. 1:**
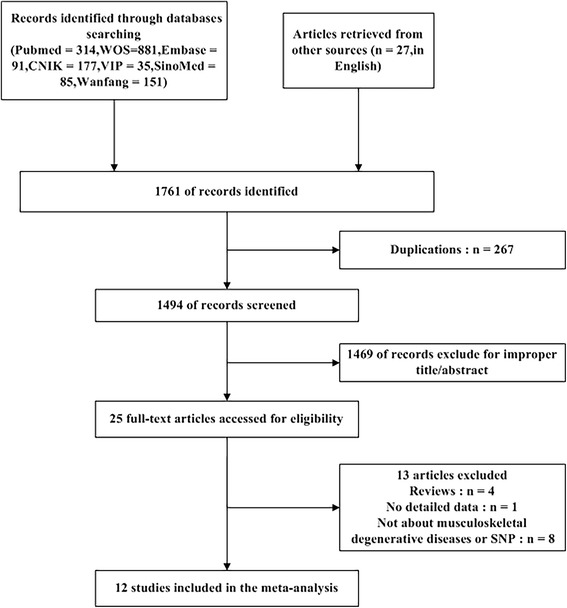
Flow diagram of studies identified, included, and excluded

A flow chart showing the exclusion/inclusion of literatures is presented as Fig. 1. The comprehensive publications search screened 1761 potentially relevant articles, of which 267 articles were excluded for duplication and 1469 articles were omitted after browsing the title and/or abstract due to obvious irrelevance to MSDD or Fas/FasL gene we studied. Eight articles were deleted because they did not study MSDD or single nucleotide polymorphisms (SNPs) unrelated to the object of our study; 1 article was excluded on account of no detailed data; and 4 articles were wiped off because they were reviews. Finally, 12 case-control studies [19, 22–32] were identified for meta-analysis based on the inclusion criteria. As shown in Table 1, 4 eligible studies for IVDD, 1 eligible study for OA, 7 eligible studies for RA. Also, 5 eligible studies for Chinese, 5 eligible studies for Caucasoid and 2 eligible studies for other race groups.

**Table 1:** Main Characteristics of Studies Included in This Meta-analysis

Study ID	Year	Enrolled Country	Ethnicity	Age Range	Gender	Diagnosis	Diagnosis by	Genotyping Method	Control Source	Matching	Cases	Controls
Lv et al. [32]	2009	China	Chinese	N/D	N/D	IVDD	N/D	PCR-seq	N/D	N/D	223	124
Zhu et al. [24]	2011	China	Chinese	30–68	both	IVDD	MRI	PCR-seq	healthy subjects	age, sex, occupation, smoking	348	215
Sun et al. [19]	2013	China	Chinese	N/D^&^	both	IVDD	MRI	PCR-seq	participants with nonspine-related problems	age, sex, race	472	528
Zhang et al. [23]	2013	China	Chinese	33–61	both	IVDD	MRI	PCR-seq	patients or participants without back pain	race, sex, age, living areas	129	132
Sezgin et al. [22]	2013	Turkey	Caucasoid	43–70	both	OA	ACR	PCR-seq	patients without OA related disease	N/D	148	102
Coakley et al. [25]	1999	UK	Caucasoid	24–87	both	RA	ACR	PCR-seq	healthy subjects	age	18	128
Huang et al. [26]	1999	Australia	Caucasoid	23–65	both	RA	ARA	PCR-seq	healthy subjects	N/D	185	86
Lee et al. [28]	2001	Korea	Korean	16–75	both	RA	ACR	PCR-seq	healthy subjects	ethnically	87	87
Mohammad- zadeh et al. [29]	2012	Iran	Caucasoid	28–59	both	RA	N/D	PCR-seq	healthy subjects	N/D	120	112
Kobak et al. [27]	2012	Turkey	N/D	19–72	both	RA	ACR	PCR-seq	healthy subjects	age, sex	101	105
Seyfi et al. [30]	2013	Turkey	Caucasoid	20–80	both	RA	ACR	PCR-seq	patients without musculoskeletal diseases	age,sex, ethnically	100	101
Zhu et al. [31]	2016	China	Chinese	40–70	both	RA	ACR	N/D	patients without RA	age, sex	839	615

*Abbreviations*: *ACR* the American college of rheumatology diagnostic criteria for RA, *ARA* American rheumatology association 1987 criteria, *IVDD* Intervertebral disc degeneration, *OA* osteoarthritis, *RA* rheumatoid arthritis, *N/D* not described, *PCR-seq* restriction analysis polymerase chain reaction sequencing. &, the author only mentioned the mean ages of the case and control groups were 36.5 and 38.7 years, respectively

As shown in Table 2, 11 eligible studies for rs1800682 with 1930 cases and 1720 controls, 6 eligible studies for rs2234767 with 1794 cases and 1909 controls, 3 eligible studies for rs5030772 with 367 cases and 313 controls and 8 eligible studies for rs763110 with 2010 cases and 2105 controls were included in this analysis. The characteristics of all the included studies are also listed in the Table 1 and Table 2, including the year, country and continent of studies, the ethnicity, age and gender of subjects, the type of MSDD, the diagnosis methods, genotyping methods, source of controls, matching items of cases and controls, the number of subjects in control/case group and Hardy-Weinberg equilibrium (HWE) in each studies. The genotype distributions for all of the control groups were consistent with the HWE, except Sezgin et al. [22]. The quality assessment of study was listed in Table 3.

**Table 2:** Distribution of genotypes among cases and controls

	Study ID	Year	Diagnosis	Ethnicity	Case Group			Control Group			P_HWE_
*FAS (CD95) site*											
*-670 G > A* *rs1800682*					GG	GA	AA	GG	GA	AA	
	Lv et al. [32]	2009	IVDD	Chinese	47	125	51	21	67	36	0.28
	Zhu et al. [24]	2011	IVDD	Chinese	57	162	129	30	96	89	0.62
	Sun et al. [19]	2013	IVDD	Chinese	74	217	181	61	265	202	0.06
	Zhang et al. [23]	2013	IVDD	Chinese	20	59	49	14	68	50	0.19
	Sezgin et al. [22]	2013	OA	Caucasoid	27	63	58	21	46	35	0.41
	Coakley et al. [25]	1999	RA	Caucasoid	4	8	6	31	61	36	0.61
	Huang et al. [26]	1999	RA	Caucasoid	32	105	48	22	44	20	0.83
	Lee et al. [28]	2001	RA	Korean	16	38	33	13	48	26	0.23
	Mohammadzadeh et al. [29]	2012	RA	Caucasoid	17	64	39	18	50	44	0.55
	Kobak et al. [27]	2012	RA	N/D	24	50	27	14	52	39	0.61
	Seyfi et al. [30]	2013	RA	Caucasoid	20	45	35	22	40	39	0.06
*−1377 G > A* *rs2234767*					GG	GA	AA	GG	GA	AA	
	Zhu et al. [24]	2011	IVDD	Chinese	121	172	55	99	92	24	0.71
	Sun et al. [19]	2013	IVDD	Chinese	218	209	45	236	248	44	0.06
	Zhang et al. [23]	2013	IVDD	Chinese	59	55	14	56	65	11	0.19
	Sezgin et al. [22]	2013	OA	Caucasoid	95	51	2	42	60	0	< 0.01
	Seyfi et al. [30]	2013	RA	Caucasoid	74	26	0	81	18	2	0.41
	Zhu et al. [31]	2016	RA	Chinese	246	284	68	389	357	85	0.82
*FASL (CD178) site*											
*IVS2nt-124* *A > G* *rs5030772*					GG	GA	AA	GG	GA	AA	
	Sezgin et al. [22]	2013	OA	Caucasoid	4	37	107	4	30	68	0.76
	Mohammadzadeh et al. [29]	2012	RA	Caucasoid	8	35	77	6	31	75	0.25
	Seyfi et al. [30]	2013	RA	Caucasoid	6	25	68	10	29	60	0.03
*−844 T > C* *rs763110*					CC	CT	TT	CC	CT	TT	
	Zhu et al. [24]	2011	IVDD	Chinese	175	148	25	131	76	8	0.46
	Sun et al. [19]	2013	IVDD	Chinese	236	188	48	308	200	20	0.07
	Zhang et al. [23]	2013	IVDD	Chinese	64	51	13	77	50	5	0.37
	Sezgin et al. [22]	2013	OA	Caucasoid	45	80	23	37	47	18	0.65
	Mohammadzadeh et al. [29]	2012	RA	Caucasoid	33	63	24	43	49	20	0.36
	Kobak et al. [27]	2012	RA	N/D	30	40	31	33	40	23	0.12
	Seyfi et al. [30]	2013	RA	Caucasoid	20	55	25	31	54	14	0.22
	Zhu et al. [31]	2016	RA	Chinese	331	228	34	453	317	51	0.65

*Abbreviations*: *HWE* Hardy–Weinberg equilibrium

**Table 3:** Quality assessment of the included articles

Study ID	year	A	B	C	D	E	F	G	H	I	J	Sum
Huang et al. [26]	1999	1	1	1	1	1	0	0	1	1	1	8
Coakley et al. [25]	1999	1	1	1	1	1	0	0	1	1	0	7
Lee et al. [28]	2001	1	1	1	1	1	0	0	1	1	0	7
Lv et al. [32]	2009	0	1	1	1	1	0	0	1	1	0	6
Zhu et al. [24]	2011	0	1	1	1	1	1	0	1	1	0	7
Mohammadzadeh et al. [29]	2011	0	1	1	1	1	0	0	1	1	0	6
Kobak et al. [27]	2012	1	1	1	1	1	0	0	1	1	0	7
Sun et al. [19]	2013	1	1	1	1	1	1	1	1	1	0	9
Zhang et al. [23]	2013	1	1	1	1	1	0	0	1	1	0	7
Sezgin et al. [22]	2013	0	0	1	1	1	1	0	1	1	0	6
Seyfi et al. [30]	2013	1	0	1	1	1	0	0	1	1	0	6
Zhu et al. [31]	2016	1	1	1	1	1	0	0	1	1	1	8

*Abbreviations*: *A* Control group, *B* Hardy–Weinberg equilibrium, *C* Case group, *D* Primer, *E* Reproducibility, *F* Blinding, *G* Power calculation, *H* Statistics, *I* Corrected statistics, *J* Independent replication, *Sum* sum of quality assessment score, *1* done, *0* undone or unclear

### Association between Fas (rs1800682) polymorphism and MSDD risk

No significant heterogeneity was noted among the studies of rs1800682 in the overall analysis, subgroup analysis leveled by diagnosis or recessive model of subgroup analysis leveled by race groups. Thus, the fixed-effects model was used for analysis in these models mentioned above. And other models used the random-effects model. However, no significant associations were found in any models for overall analysis.

**Fig. 2:**
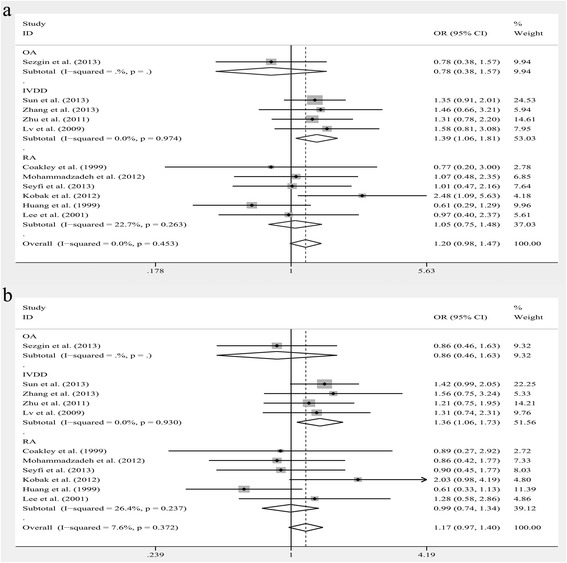
The associations of Fas (rs1800682) with MSDD leveled by diagnosis in different genetic models. **a** Homozygote model (GG vs. AA). **b** Recessive model (GG vs. GA + AA)

**Fig. 3:**
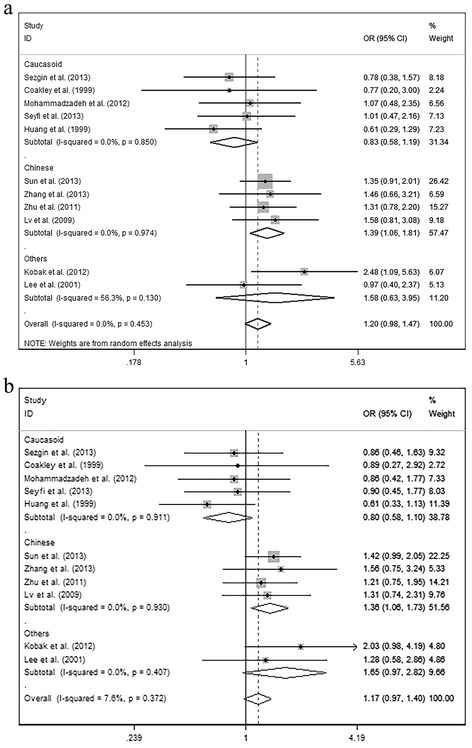
The associations of Fas (rs1800682) with MSDD leveled by race groups in different genetic models. **a** Homozygote model (GG vs. AA). **b** Recessive model (GG vs. GA + AA)

The results of subgroup analyses leveled by diagnosis were listed below: For OA subgroup, no significant relationship was found in any models. For IVDD subgroup, significant associations were noted in GG vs. AA, OR = 1.388, 95% CI: 1.062–1.812, *P* = 0.016; in GG vs. GA + AA, OR = 1.357, 95% CI: 1.063–1.731, *P* = 0.014 (Fig. 2). However, no significant relationship was observed in other models. For RA subgroup, no significant relationship was found in any models. (Additional file 1: Table S1) The results of subgroup analyses leveled by race groups were listed below: For Caucasoid subgroup, no significant relationship was found in any models. For Chinese subgroup, significant associations were noted in GG vs. AA, OR = 1.388, 95% CI: 1.062–1.812, *P* = 0.016; in GG vs. GA + AA, OR = 1.357, 95% CI: 1.063–1.731, *P* = 0.014 (Fig. 3). However, no significant relationship was observed in other models. (Additional file 2: Table S2).

### Association between Fas (rs2234767) polymorphism and MSDD risk

Significant heterogeneity was observed among the studies of rs2234767 in the allele and recessive models for overall analysis and subgroup analysis leveled by diagnosis and allele, heterozygote and recessive models of subgroup analysis leveled by race groups. Thus, the random-effects model was chosen to assess the connection between rs2234767 polymorphism and MSDD risk in these models mentioned above. And other models used the fixed-effects model. Significant associations were noted in GG vs. AA, OR = 0.771, 95% CI: 0.608–0.976, *P* = 0.031. However, no significant relationship was observed in other models.

**Fig. 4:**
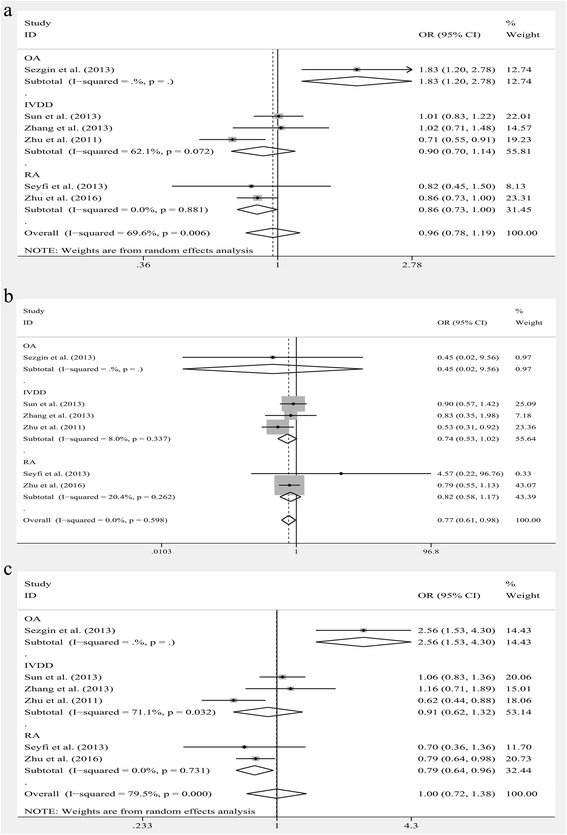
The associations of Fas (rs2234767) with MSDD leveled by diagnosis in different genetic models. **a** Allele model (G vs. A). **b** Homozygote model (GG vs. AA). **c** Recessive model (GG vs. GA + AA)

**Fig. 5:**
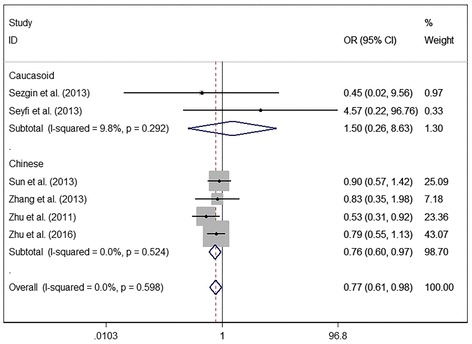
The associations of Fas (rs2234767) with MSDD leveled by race groups in Homozygote model (GG vs. AA)

The results of subgroup analyses leveled by diagnosis were showed below: For OA subgroup, significant associations were found in G vs. A, OR = 1.826, 95% CI: 1.199–2.779, *P* = 0.005; in GG vs. GA + AA, OR = 2.561, 95% CI: 1.525–4.299, *P* = 0.000 (Fig. 4). However, no significant relationship was observed in other models. For IVDD subgroup, no significant relationship was found in any models. For RA subgroup, significant associations were explored in G vs. A, OR = 0.855, 95% CI: 0.734–0.996, *P* = 0.044; in GG vs. GA + AA, OR = 0.785, 95% CI: 0.641–0.961, *P* = 0.019 (Fig. 4). However, no significant relationship was observed in other models. (Additional file 1: Table S1) The results of subgroup analyses leveled by race groups were showed below: For Caucasoid subgroup no significant relationship was observed in any models. For Chinese subgroup significant associations was explored in GG vs. AA, OR = 0.761, 95% CI: 0.599–0.966, *P* = 0.025 (Fig. 5). However, no significant relationship was observed in other models. (Additional file 2: Table S2).

### Association between FasL (rs5030772) polymorphism and MSDD risk

Significant heterogeneity was explored among the studies of rs5030772 in the allele model of overall and subgroup leveled by diagnosis analysis. Thus, the random-effects model was used to evaluate the association between rs5030772 polymorphism and MSDD risk in allele model. The other models used the fixed-effects model. However, no significant associations were observed in any models for overall analysis. The results of subgroup analyses leveled by diagnosis were listed below: For OA subgroup, no significant relationship was found in any models. For RA subgroup, no significant relationship was found in any models. (Additional file 3: Table S3) The results of subgroup analyses leveled by race groups were listed below: For Caucasoid subgroup, no significant relationship was found in any models. (Additional file 4: Table S4).

### Association between FasL (rs763110) polymorphism and MSDD risk

**Fig. 6:**
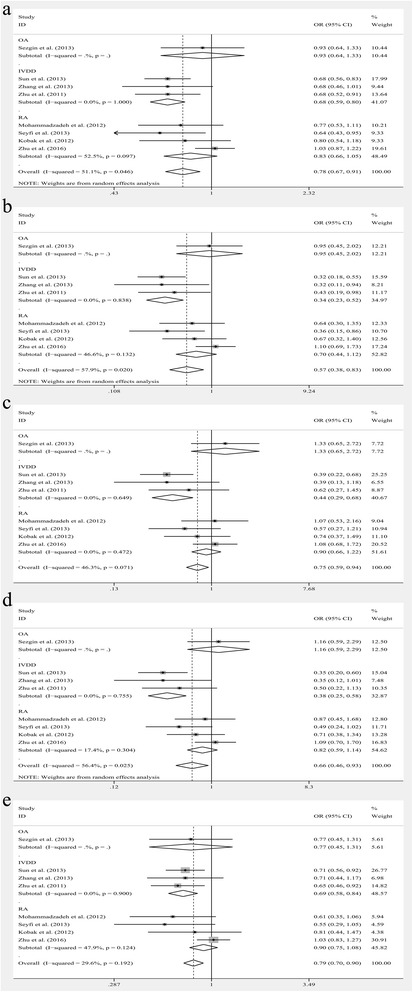
The associations of FasL (rs763110) with MSDD leveled by diagnosis in different genetic models. **a** Allele model (C vs. T). **b** Homozygote model (CC vs. TT). **c** Heterozygote model (CT vs. TT). **d** Dominant model (CC + CT vs. TT). **e** Recessive model (CC vs. CT + TT)

Significant heterogeneity was observed among the studies of rs763110 in the allele, homozygote and dominant models for overall analysis and subgroup analyses stratified by diagnosis and all the models stratified by race groups. So the random-effects model was applied to assess the association between rs763110 polymorphism and MSDD risk in models mentioned above. Other models used the fixed-effects model. Significant associations were noted in all models: in the allele model, C vs. T, OR = 0.780, 95% CI: 0.671–0.907, *P* = 0.001; in the homozygote model, CC vs. TT, OR = 0.565, 95% CI: 0.383–0.834, *P* = 0.004; in the heterozygote model, CT vs. TT, OR = 0.746, 95% CI: 0.591–0.946, *P* = 0.013; in the dominant model, CC + CT vs. TT, OR = 0.656, 95% CI: 0.461–0.934, *P* = 0.019; and in recessive model, CC vs. CT + TT, OR = 0.794, 95% CI: 0.700–0.901, *P* = 0.000 (Fig. 6).

**Fig. 7:**
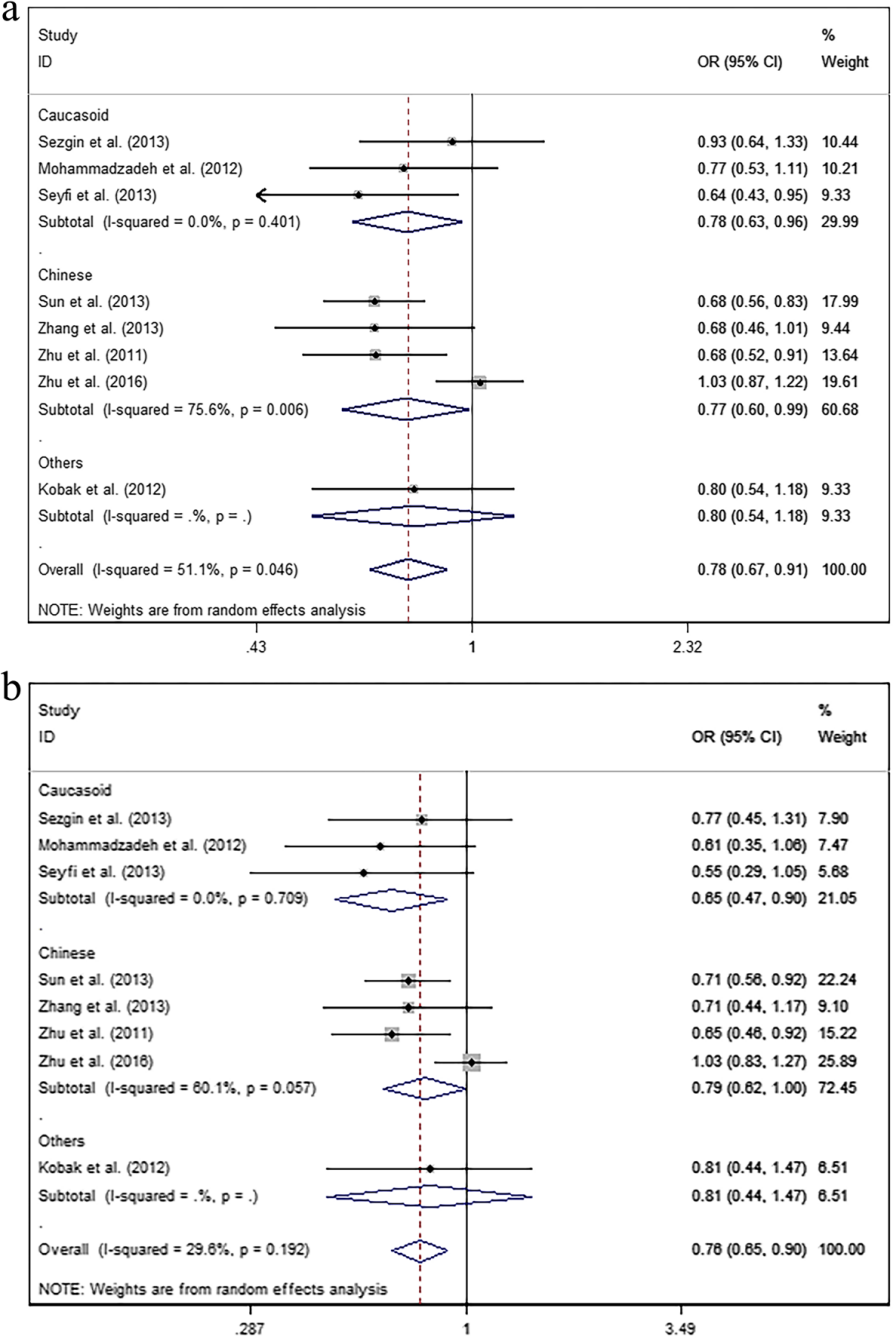
The associations of FasL (rs763110) with MSDD leveled by race groups in different genetic models. **a** Allele model (C vs. T). **b** Recessive model (CC vs. CT + TT)

The results of subgroup analyses leveled by diagnosis were showed below: For OA subgroup, no significant relationship was found in any models. For IVDD subgroup, significant associations were explored in C vs. T, OR = 0.684, 95% CI: 0.588–0.795, *P* = 0.000; in CC vs. TT, OR = 0.344, 95% CI: 0.226–0.525, *P* = 0.000; in CT vs. TT, OR = 0.442, 95% CI: 0.288–0.679, *P* = 0.000; in CC + CT vs. TT, OR = 0.382, 95% CI: 0.253–0.577, *P* = 0.001; in CC vs. CT + TT, OR = 0.694, 95% CI: 0.576–0.837, *P* = 0.001 (Fig. 6). For RA subgroup, no significant relationship was found in any models. (Additional file 3: Table S3) The results of subgroup analyses leveled by race groups were showed below: For Caucasoid subgroup, significant associations were explored in C vs. T, OR = 0.777, 95% CI: 0.626–0.964, *P* = 0.022; in CC vs. CT + TT, OR = 0.647, 95% CI: 0.465–0.901, *P* = 0.010 (Fig. 7). However, no significant relationship was observed in other models. For Chinese subgroup, significant associations were explored in C vs. T, OR = 0.772, 95% CI: 0.603–0.989, *P* = 0.041; in CC vs. CT + TT, OR = 0.786, 95% CI: 0.618–0.998, *P* = 0.048 (Fig. 7). However, no significant relationship was observed in other models. (Additional file 4: Table S4).

### Sensitivity analysis

Sensitivity analysis was performed to assess the influence set by one study on the pooled ORs for Fas (rs1800682, rs2234767) and FasL (rs5030772, rs763110) polymorphism by deleting one study each turn in every genetic model.

We observed that the pooled ORs significantly differed when we deleted Huang et al. [26] in homozygote model (GG vs. AA, OR = 1.269, 95% CI: 1.030–1.565, *P* = 0.025) and in recessive model (GG vs. GA + AA, OR = 1.240, 95% CI: 1.026–1.499, *P* = 0.026) for Fas (rs1800682) site. We also noted that the overall ORs significantly changed when we deleted Zhu et al. (published in 2011) [24] in homozygote model (GG vs. AA, OR = 0.843, 95% CI: 0.647–1.098, *P* = 0.204) and Zhu et al. (published in 2016) [31] in homozygote model (GG vs. AA, OR = 0.755, 95% CI: 0.550–1.037, *P* = 0.082) for Fas (rs2234767) site. We found that the pooled ORs significantly differed when we deleted Sun et al. 19] in dominant model (GG + GA vs. AA, OR = 0.760, 95%CI: 0.560–1.031, *P* = 0.078) and Seyfi et al. [30] in dominant model (GG + GA vs. AA, OR = 0.679, 95% CI: 0.459–1.005, *P* = 0.053) for FasL (rs763110) site as well. However, there was no change in the significance of results in any models for FasL (rs5030772) site.

### Publication bias

**Fig. 8:**
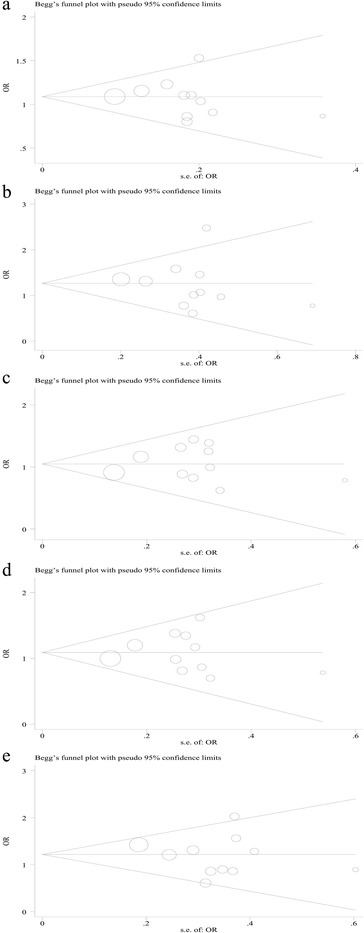
Begg’s funnel plot of publication bias for the association of FAS (rs1800682) polymorphism with MSDD in different genetic models. **a** Allele model (G vs. A). **b** Homozygote model (GG vs. AA). **c** Heterozygote model (GA vs. AA). **d** Dominant model (GG + GA vs. AA). **e** Recessive model (GG vs. GA + AA). Each point represents a separate study for the indicated association

The Begg funnel plot (Fig. 8) and the Egger’s test were conducted to evaluate the publication bias in selected literature. No evidence of publication bias was noted in this study for Fas rs1800682 (Begg’s test: *P* = 0.436, Egger’s test: *P* = 0.576 for allele model; Begg’s test *P* = 0.640, Egger’s test *P* = 0.609 for homozygote model; Begg’s test *P* = 0.876, Egger’s test *P* = 0.694 for heterozygote model; Begg’s test: *P* = 0.640, Egger’s test: *P* = 0.965 for dominant model; Begg’s test: *P* = 1.000, Egger’s test: *P* = 0.508 for recessive model).(Table 4) Because of the limited number (below 10) of studies included in Fas (rs2234767) and FasL (rs5030772, rs763110), publication bias was not evaluated in these sites.

**Table 4:** Publication bias tests for association of the Fas (rs1800682) polymorphisms with musculoskeletal degenerative diseases

Comparisons	Egger’s test			Begg’s test
	*t*	95% CI	*P* value	*P* value
allele model	−0.58	(−2.91,1.72)	0.576	0.436
homozygote model	−0.53	(−3.70,2.29)	0.609	0.640
heterozygote model	0.41	(−1.56,2.24)	0.694	0.876
dominant model	0.05	(−2.03,2.11)	0.965	0.640
recessive model	−0.69	(−3.82,2.03)	0.508	1.000

*Abbreviations*: *CI* confidence interval

## Discussion

MSDD are common and one of the most clinically vital somatic disorders. A large number of genetic factors have been discovered among the crucial causes of IVDD [39], RA [40] and OA [41, 42] Several studies have reported the Fas/FasL genetic polymorphisms to be related to MSDD, but with conflicting results. In order to offer insight into the connection between Fas/FasL gene and diseases, large sample studies about predisposing gene polymorphisms are required. A meta-analysis, critically reviewing 11 studies on Fas (rs1800682), 6 studies on Fas (rs2234767), 3 studies on FasL (rs5030772) and 8 studies on FasL (rs763110), was performed to assess the association of Fas/FasL genetic polymorphisms with the risk of MSDD. Its strength came from the accumulation of published data, offering more information to evaluate significant differences.

In current meta-analysis, the main findings were that the G allele of Fas (rs2234767) was linked to a decreased risk of MSDD only in homozygote model and the T allele of FasL (rs763110) was associated with a reduced risk of MSDD in all of the comparison models. Besides that, subgroup analyses leveled by diagnosis suggested that the G allele of Fas (rs1800682) was associated with an increased risk of IVDD in homozygote and recessive models. The G allele of Fas (rs2234767) was linked to a decreased risk of RA but an enhanced risk of OA in allele and recessive models. In addition, the T allele of FasL (rs763110) was correlated with a reduced risk of IVDD in all of models. However, no relationship was found between FasL (rs5030772) and these three types of MSDD in any models. In addition, subgroup analyses leveled by race groups showed that the G allele of Fas (rs1800682) was associated with an increased risk of MSDD in homozygote and recessive models only in Chinese people. The G allele of Fas (rs2234767) was linked to a decreased risk of MSDD in homozygote model for Chinese people. What’s more, the T allele of FasL (rs763110) was correlated with a reduced risk of MSDD in allele and recessive models for both Caucasoid and Chinese race groups. However, no relationship was found between FasL (rs5030772) and these two race groups of MSDD in any models. Our results have several differences with the previous meta-analyses of RA recently published [31, 33]. Compared with Lee et al. 33], we observed no significance between FasL rs763110 and RA but it got an opposite result. A possible explanation for this phenomenon is that we include one more study with large participants, Zhu et al. (published in 2016) [31] Although Lee et al. [33] got the same result with us in Fas (rs1800682) site, it has some errors in data extraction of articles as mentioned above. We corrected the mistakes and analyzed again. Compared with Zhu et al. (published in 2016) [31], the small differences of result in Fas (rs2234767) for RA might be due to a new study [30] that we added in analysis. Furthermore, these Fas/FasL polymorphisms influencing the risk of MSDD can be explained partly by that these mutations can remarkably alter the percent of resident cells in tissues, such as disc cells in IVD and T-lymphocyte in synovial tissue, gradually causing occurrence of these MSDD [17, 19]. In addition, the same single nucleotide polymorphism exerted disproportionate levels of influence on different MSDD. These might be interpreted by various histology constitutions among IVDD, OA and RA. Finally, significant heterogeneity was noted in allele and recessive models of Fas (rs2234767), in allele model of FasL (rs5030772) and in allele, homozygote and dominant models of FasL (rs763110). Discrepancy among three types of MSDD might contribute to these heterogeneities. Other factors, such as ethnicity, sex distribution, occupation and etc. might also be potential sources of heterogeneity.

What’s more, the genotype distributions of controls in all of models were in accordance with HWE, except Sezgin et al. [22] in Fas (rs2234767) and Seyfi et al. [30] in FasL (rs5030772). However, the association was not significant change when ruled out these two studies by excluding one at a time. The quality assessment indicated that the enrolled studies were credible. No evidences of publication bias were observed by either Begg’s or Egger’s test in Fas (rs1800682). In order to analyze the stability of the overall results, sensitivity analysis by deleting each included studies was managed in this meta-analysis. Only one or two studies influenced the result of analysis in some models for Fas (rs1800682, rs2234767) and FasL (rs763110) and no study affects the results for FasL (rs5030772), suggesting the results were reliable in some extents. But more studies still need to be conducted in order to verify the outcome of the current meta-analysis. Overall, the results of this meta-analysis are credible and stable to a certain degree.

There are some limitations in the present study. Firstly, only three studies for FasL rs5030772 site were included in analysis and only one article was screened out for OA subgroup analysis due to shortage of original studies. Secondly, the heterogeneity was a little bit high (*I*^*2*^ > 50%) in some models for overall analyses, leading to a cautious acceptance of the results. So we performed subgroup analyses stratified by diagnosis to make the result more credible. What’s more, some of the included articles did not match the confounding factors such as age, sex and ethnicity between case group and control group. And different factors for matching might also increase the probability of residual confounding. Such confounding factors might influence the final results. However, this meta-analysis has some strength. For example, to our best knowledge, this is the most comprehensive meta-analysis focused on the association of Fas/FasL gene polymorphism with the susceptibility of MSDD, including OA, IVDD and RA. Several strategies and rigid criteria were set to assess the methodological quality of each study; all of the included studies possessed high or moderate qualities.

## Conclusions

In summary, the current meta-analysis suggested that Fas (rs1800682) and FasL (rs763110) polymorphism were associated with the susceptibility of IVDD. Fas (rs2234767) was correlated to the risk of OA and RA. Fas (rs1800682) and Fas (rs2234767) are more likely to be associated with MSDD for Chinese people. FasL (rs763110) is related to the progression of MSDD for both Caucasoid and Chinese race groups. However, FasL (rs5030772) might not be associated with MSDD. Because of the above-mentioned limitations, large-scale studies, including larger populations and considering more confounding factors, are required to verify the outcomes of this meta-analysis.

## Additional files

Additional file1: **Table S1.** Summary of meta-analysis for the association of Fas rs1800682 and rs2234767 polymorphisms with musculoskeletal degenerative diseases leveled by diagnosis. (DOCX 25 kb)

Additional file 2: **Table S2.** Summary of meta-analysis for the association of FAS rs1800682 and rs2234767 polymorphisms with musculoskeletal degenerative diseases leveled by race groups. (DOCX 23 kb)

Additional file 3: **Table S3.** Summary of meta-analysis for the association of FasL rs5030772 and rs763110 polymorphisms with musculoskeletal degenerative diseases leveled by diagnosis. (DOCX 24 kb)

Additional file 4: **Table S4.** Summary of meta-analysis for the association of FASL rs5030772 and rs763110 polymorphisms with musculoskeletal degenerative diseases leveled by race groups. (DOCX 22 kb)

## Abbreviations

CI: Confidence interval
CNKI: China National Knowledge Infrastructure
FasL: Fas ligand
HWE: Hardy-Weinberg equilibrium
IVD: Intervertebral disc
IVDD: Intervertebral disc degeneration
MSDD: Musculoskeletal degenerative diseases
OA: Osteoarthritis
OR: Odds ratio
RA: Rheumatoid arthritis
SNPs: Single nucleotide polymorphisms
WOS: Web of Science
