# Supplementary material for: Association between Fas/FasL gene polymorphism and musculoskeletal degenerative diseases: a meta-analysis
Source: BMC Musculoskelet Disord. 2018 May 7;19:137. doi: 10.1186/s12891-018-2057-z (PMC5938814; doi:10.1186/s12891-018-2057-z)
Supplement: Supplementary file 1 — Table S1. Summary of meta-analysis for the association of Fas rs1800682 and rs2234767 polymorphisms with musculoskeletal degenerative diseases leveled by diagnosis. (DOCX 25 kb) [file 12891_2018_2057_MOESM1_ESM.docx]

| **TABLE S1. Summary of meta-analysis for the association of Fas rs1800682 and rs2234767 polymorphisms with musculoskeletal degenerative diseases leveled by diagnosis** | | | | | | | | | | | |
| --- | --- | --- | --- | --- | --- | --- | --- | --- | --- | --- | --- |
| **Genetic Model** | **Stratifications** | **N** |  | **ORs Analysis** | |  | **Heterogeneity Analysis** | | |  | **M** |
|  |  |  |  | **polled ORs (95% CI)** | **P value** |  | **χ2** | **P_heterogeneity_** | ***I^2^* (%)** |  |  |
| Fas rs1800682 |  |  |  |  |  |  |  |  |  |  |  |
|  |  |  |  |  |  |  |  |  |  |  |  |
| allele model | Overall | 11 |  | 1.077 (0.978,1.186) | 0.131 |  | 9.15 | 0.518 | 0.00% |  | Fixed |
|  | OA | 1 |  | 0.862 (0.600,1.237) | 0.420 |  | 0.00 | - | 0.00% |  |  |
|  | IVDD | 4 |  | 1.129 (0.997,1.278) | 0.056 |  | 0.48 | 0.922 | 0.00% |  |  |
|  | RA | 6 |  | 1.037 (0.876,1.226) | 0.675 |  | 6.46 | 0.264 | 22.60% |  |  |
| homozygote model | Overall | 11 |  | 1.203 (0.984,1.471) | 0.071 |  | 9.86 | 0.453 | 0.00% |  | Fixed |
|  | OA | 1 |  | 0.776 (0.382,1.575) | 0.482 |  | 0.00 | - | 0.00% |  |  |
|  | IVDD | 4 |  | 1.388 (1.062,1.812) | 0.016 |  | 0.22 | 0.974 | 0.00% |  |  |
|  | RA | 6 |  | 1.054 (0.749,1.482) | 0.762 |  | 6.47 | 0.263 | 22.70% |  |  |
| heterozygote model | Overall | 11 |  | 1.026 (0.884,1.189) | 0.739 |  | 7.95 | 0.634 | 0.00% |  | Fixed |
|  | OA | 1 |  | 0.826 (0.469,1.456) | 0.509 |  | 0.00 | - | 0.00% |  |  |
|  | IVDD | 4 |  | 1.016 (0.842,1.225) | 0.872 |  | 2.34 | 0.506 | 0.00% |  |  |
|  | RA | 6 |  | 1.026 (0.884,1.189) | 0.493 |  | 4.79 | 0.442 | 0.00% |  |  |
| dominant model | Overall | 11 |  | 1.066 (0.926,1.226) | 0.374 |  | 8.14 | 0.615 | 0.00% |  | Fixed |
|  | OA | 1 |  | 0.811 (0.479,1.371) | 0.433 |  | 0.00 | - | 0.00% |  |  |
|  | IVDD | 4 |  | 1.088 (0.910,1.299) | 0.355 |  | 1.79 | 0.617 | 0.00% |  |  |
|  | RA | 6 |  | 1.090 (0.847,1.402) | 0.503 |  | 5.23 | 0.388 | 4.40% |  |  |
| recessive model | Overall | 11 |  | 1.168 (0.975,1.400) | 0.092 |  | 10.82 | 0.372 | 7.60% |  | Fixed |
|  | OA | 1 |  | 0.861 (0.456,1.626) | 0.644 |  | 0.00 | - | 0.00% |  |  |
|  | IVDD | 4 |  | 1.357 (1.063,1.731) | 0.014 |  | 0.45 | 0.930 | 0.00% |  |  |
|  | RA | 6 |  | 0.993 (0.735,1.341) | 0.962 |  | 6.79 | 0.237 | 26.40% |  |  |
| Fas rs2234767 |  |  |  |  |  |  |  |  |  |  |  |
|  |  |  |  |  |  |  |  |  |  |  |  |
| allele model | Overall | 6 |  | 0.964 (0.784,1.185) | 0.728 |  | 16.45 | 0.006 | 69.60% |  | Random |
|  | OA | 1 |  | 1.826 (1.199,2.779) | 0.005 |  | 0.00 | - | 0.00% |  |  |
|  | IVDD | 3 |  | 0.896 (0.702,1.143) | 0.377 |  | 5.27 | 0.072 | 62.10% |  |  |
|  | RA | 2 |  | 0.855 (0.734,0.996) | 0.044 |  | 0.02 | 0.881 | 0.00% |  |  |
| homozygote model | Overall | 6 |  | 0.771 (0.608,0.976) | 0.031 |  | 3.67 | 0.598 | 0.00% |  | Fixed |
|  | OA | 1 |  | 0.449 (0.021,9.564) | 0.608 |  | 0.00 | - | 0.00% |  |  |
|  | IVDD | 3 |  | 0.738 (0.535,1.019) | 0.065 |  | 2.17 | 0.337 | 8.00% |  |  |
|  | RA | 2 |  | 0.819 (0.576,1.165) | 0.267 |  | 1.26 | 0.262 | 20.40% |  |  |
| heterozygote model | Overall | 6 |  | 0.885 (0.699,1.120) | 0.308 |  | 3.88 | 0.566 | 0.00% |  | Fixed |
|  | OA | 1 |  | 0.170 (0.008,3.627) | 0.257 |  | 0.00 | - | 0.00% |  |  |
|  | IVDD | 3 |  | 0.797 (0.577,1.101) | 0.169 |  | 0.2 | 0.907 | 0.00% |  |  |
|  | RA | 2 |  | 1.033 (0.727,1.467) | 0.856 |  | 1.55 | 0.213 | 35.40% |  |  |
| dominant model | Overall | 6 |  | 0.826 (0.660,1.034) | 0.096 |  | 2.75 | 0.739 | 0.00% |  | Fixed |
|  | OA | 1 |  | 0.286 (0.014,6.017) | 0.420 |  | 0.00 | - | 0.00% |  |  |
|  | IVDD | 3 |  | 0.769 (0.566,1.045) | 0.093 |  | 0.56 | 0.757 | 0.00% |  |  |
|  | RA | 2 |  | 0.917 (0.656,1.280) | 0.610 |  | 1.24 | 0.266 | 19.20% |  |  |
| recessive model | Overall | 6 |  | 0.996 (0.719,1.379) | 0.980 |  | 24.39 | 0.000 | 79.50% |  | Random |
|  | OA | 1 |  | 2.561 (1.525,4.299) | 0.000 |  | 0.00 | - | 0.00% |  |  |
|  | IVDD | 3 |  | 0.908 (0.622,1.324) | 0.615 |  | 6.91 | 0.032 | 71.10% |  |  |
|  | RA | 2 |  | 0.785 (0.641,0.961) | 0.019 |  | 0.12 | 0.731 | 0.00% |  |  |
| *Abbreviations: M, model used for meta-analysis; CI, confidence interval; ORs, odds ratios; N, number of studies included in each analysis.* | | | | | | | | | | | |
