# Supplementary material for: Association between Fas/FasL gene polymorphism and musculoskeletal degenerative diseases: a meta-analysis
Source: BMC Musculoskelet Disord. 2018 May 7;19:137. doi: 10.1186/s12891-018-2057-z (PMC5938814; doi:10.1186/s12891-018-2057-z)
Supplement: Supplementary file 2 — Table S2. Summary of meta-analysis for the association of FAS rs1800682 and rs2234767 polymorphisms with musculoskeletal degenerative diseases leveled by race groups. (DOCX 23 kb) [file 12891_2018_2057_MOESM2_ESM.docx]

| **TABLE S2. Summary of meta-analysis for the association of FAS rs1800682 and rs2234767 polymorphisms with musculoskeletal degenerative diseases leveled by race groups** | | | | | | | | | | | |
| --- | --- | --- | --- | --- | --- | --- | --- | --- | --- | --- | --- |
| **Genetic Model** | **Stratifcations** | **N** |  | **ORs ananlysis** | |  | **Heterogeneity Analysis** | | |  | **M** |
|  |  |  |  | **polled ORs (95% CI)** | **P value** |  | **χ2** | **P_heterogeneity_** | **I^2^ (%)** |  |  |
| FAS rs1800682 |  |  |  |  |  |  |  |  |  |  |  |
|  |  |  |  |  |  |  |  |  |  |  |  |
| allele model | Overall | 11 |  | 1.077 (0.978,1.186) | 0.132 |  | 9.15 | 0.518 | 0.00% |  | Random |
|  | Caucasoid | 5 |  | 0.933 (0.780,1.117) | 0.452 |  | 1.99 | 0.738 | 0.00% |  |  |
|  | Chinese | 4 |  | 1.129 (0.997,1.278) | 0.056 |  | 0.48 | 0.922 | 0.00% |  |  |
|  | Others | 2 |  | 1.189 (0.714,1.980) | 0.507 |  | 3.1 | 0.078 | 67.80% |  |  |
| homozygote model | Overall | 11 |  | 1.202 (0.982,1.472) | 0.075 |  | 9.86 | 0.453 | 0.00% |  | Random |
|  | Caucasoid | 5 |  | 0.832 (0.580,1.195) | 0.319 |  | 1.36 | 0.85 | 0.00% |  |  |
|  | Chinese | 4 |  | 1.388 (1.062,1.812) | 0.016 |  | 0.22 | 0.974 | 0.00% |  |  |
|  | Others | 2 |  | 1.577 (0.629,3.949) | 0.331 |  | 2.29 | 0.13 | 56.30% |  |  |
| heterozygote model | Overall | 11 |  | 1.026 (0.884,1.190) | 0.737 |  | 7.95 | 0.634 | 0.00% |  | Random |
|  | Caucasoid | 5 |  | 1.079 (0.810,1.439) | 0.602 |  | 2.45 | 0.654 | 0.00% |  |  |
|  | Chinese | 4 |  | 1.016 (0.842,1.225) | 0.869 |  | 2.34 | 0.506 | 0.00% |  |  |
|  | Others | 2 |  | 0.939 (0.429,2.057) | 0.875 |  | 2.94 | 0.086 | 66.00% |  |  |
| dominant model | Overall | 11 |  | 1.066 (0.926,1.227) | 0.372 |  | 8.14 | 0.615 | 0.00% |  | Random |
|  | Caucasoid | 5 |  | 1.008 (0.770,1.319) | 0.956 |  | 2.48 | 0.647 | 0.00% |  |  |
|  | Chinese | 4 |  | 1.088 (0.911,1.300) | 0.352 |  | 1.79 | 0.617 | 0.00% |  |  |
|  | Others | 2 |  | 1.071 (0.469,2.444) | 0.871 |  | 3.64 | 0.056 | 72.50% |  |  |
| recessive model | Overall | 11 |  | 1.168 (0.975,1.400) | 0.092 |  | 10.82 | 0.372 | 7.60% |  | Fixed |
|  | Caucasoid | 5 |  | 0.797 (0.580,1.096) | 0.162 |  | 0.99 | 0.911 | 0.00% |  |  |
|  | Chinese | 4 |  | 1.357 (1.063,1.731) | 0.014 |  | 0.45 | 0.930 | 0.00% |  |  |
|  | Others | 2 |  | 1.652 (0.967,2.822) | 0.066 |  | 0.69 | 0.407 | 0.00% |  |  |
|  |  |  |  |  |  |  |  |  |  |  |  |
| FAS rs2234767 |  |  |  |  |  |  |  |  |  |  |  |
|  |  |  |  |  |  |  |  |  |  |  |  |
| allele model | Overall | 6 |  | 0.964 (0.784,1.185) | 0.728 |  | 16.45 | 0.006 | 69.60% |  | Random |
|  | Caucasoid | 2 |  | 1.260 (0.575,2.762) | 0.564 |  | 4.57 | 0.033 | 78.10% |  |  |
|  | Chinese | 4 |  | 0.883 (0.759,1.028) | 0.109 |  | 5.54 | 0.137 | 45.80% |  |  |
| homozygote model | Overall | 6 |  | 0.771 (0.608,0.976) | 0.031 |  | 3.67 | 0.598 | 0.00% |  | Fixed |
|  | Caucasoid | 2 |  | 1.495 (0.259,8.632) | 0.653 |  | 1.11 | 0.292 | 9.80% |  |  |
|  | Chinese | 4 |  | 0.761 (0.599,0.966) | 0.025 |  | 2.24 | 0.524 | 0.00% |  |  |
| heterozygote model | Overall | 6 |  | 0.883 (0.696,1.120) | 0.306 |  | 3.88 | 0.566 | 0.00% |  | Random |
|  | Caucasoid | 2 |  | 1.096 (0.028,42.788) | 0.961 |  | 2.84 | 0.092 | 64.80% |  |  |
|  | Chinese | 4 |  | 0.881 (0.694,1.119) | 0.299 |  | 1.01 | 0.798 | 0.00% |  |  |
| dominant model | Overall | 6 |  | 0.826 (0.660,1.034) | 0.096 |  | 2.75 | 0.739 | 0.00% |  | Fixed |
|  | Caucasoid | 2 |  | 1.211 (0.230,6.368) | 0.821 |  | 1.71 | 0.192 | 41.40% |  |  |
|  | Chinese | 4 |  | 0.820 (0.654,1.029) | 0.087 |  | 0.93 | 0.819 | 0.00% |  |  |
| recessive model | Overall | 6 |  | 0.996 (0.719,1.379) | 0.980 |  | 24.39 | 0.000 | 79.50% |  | Random |
|  | Caucasoid | 2 |  | 1.365 (0.384,4.844) | 0.63 |  | 9.08 | 0.003 | 89.00% |  |  |
|  | Chinese | 4 |  | 0.869 (0.679,1.112) | 0.265 |  | 7.98 | 0.046 | 62.40% |  |  |
| *Abbreviations: M, model used for meta-analysis; CI, confidence interval; ORs, odds ratios; N, number of studies included in each analysis.* | | | | | | | | | | | |
|  |  |  |  |  |  |  |  |  |  |  |  |
