# Supplementary material for: Association between Fas/FasL gene polymorphism and musculoskeletal degenerative diseases: a meta-analysis
Source: BMC Musculoskelet Disord. 2018 May 7;19:137. doi: 10.1186/s12891-018-2057-z (PMC5938814; doi:10.1186/s12891-018-2057-z)
Supplement: Supplementary file 3 — Table S3. Summary of meta-analysis for the association of FasL rs5030772 and rs763110 polymorphisms with musculoskeletal degenerative diseases leveled by diagnosis. (DOCX 24 kb) [file 12891_2018_2057_MOESM3_ESM.docx]

| **TABLE S3. Summary of meta-analysis for the association of FasL rs5030772 and rs763110 polymorphisms with musculoskeletal degenerative diseases leveled by diagnosis** | | | | | | | | | | | |
| --- | --- | --- | --- | --- | --- | --- | --- | --- | --- | --- | --- |
| **Genetic Model** | **Stratifications** | **N** |  | **ORs Analysis** | |  | **Heterogeneity Analysis** | | |  | **M** |
|  |  |  |  | **polled ORs (95% CI)** | **P value** |  | **χ2** | **P_heterogeneity_** | ***I^2^* (%)** |  |  |
| FasL rs5030772 |  |  |  |  |  |  |  |  |  |  |  |
|  |  |  |  |  |  |  |  |  |  |  |  |
| allele model | Overall | 3 |  | 0.861 (0.644,1.152) | 0.314 |  | 2.30 | 0.316 | 13.20% |  | Random |
|  | OA | 1 |  | 0.783 (0.487,1.258) | 0.312 |  | 0.00 | - | 0.00% |  |  |
|  | RA | 2 |  | 0.897 (0.557,1.444) | 0.655 |  | 2.07 | 0.15 | 51.70% |  |  |
| homozygote model | Overall | 3 |  | 0.772 (0.397,1.502) | 0.446 |  | 1.40 | 0.496 | 0.00% |  | Fixed |
|  | OA | 1 |  | 0.636 (0.154,2.626) | 0.531 |  | 0.00 | - | 0.00% |  |  |
|  | RA | 2 |  | 0.815 (0.384,1.730) | 0.594 |  | 1.31 | 0.253 | 23.50% |  |  |
| heterozygote model | Overall | 3 |  | 0.875 (0.622,1.232) | 0.445 |  | 0.93 | 0.629 | 0.00% |  | Fixed |
|  | OA | 1 |  | 0.784 (0.444,1.385) | 0.402 |  | 0.00 | - | 0.00% |  |  |
|  | RA | 2 |  | 0.931 (0.607,1.428) | 0.743 |  | 0.7 | 0.401 | 0.00% |  |  |
| dominant model | Overall | 3 |  | 0.856 (0.621,1.180) | 0.344 |  | 1.62 | 0.445 | 0.00% |  | Fixed |
|  | OA | 1 |  | 0.766 (0.444,1.324) | 0.340 |  | 0.00 | - | 0.00% |  |  |
|  | RA | 2 |  | 0.907 (0.610,1.349) | 0.631 |  | 1.38 | 0.240 | 27.60% |  |  |
| recessive mode | Overall | 3 |  | 0.800 (0.414,1.546) | 0.507 |  | 1.10 | 0.576 | 0.00% |  | Fixed |
|  | OA | 1 |  | 0.681 (0.166,2.786) | 0.593 |  | 0.00 | - | 0.00% |  |  |
|  | RA | 2 |  | 0.837 (0.397,1.761) | 0.638 |  | 1.04 | 0.576 | 3.40% |  |  |
| FasL rs763110 |  |  |  |  |  |  |  |  |  |  |  |
|  |  |  |  |  |  |  |  |  |  |  |  |
| allele model | Overall | 8 |  | 0.780 (0.671,0.907) | 0.001 |  | 14.31 | 0.046 | 51.10% |  | Random |
|  | OA | 1 |  | 0.925 (0.644,1.329) | 0.675 |  | 0.00 | - | 0.00% |  |  |
|  | IVDD | 3 |  | 0.684 (0.588,0.795) | 0.000 |  | 0.00 | 1.000 | 0.00% |  |  |
|  | RA | 4 |  | 0.834 (0.662,1.051) | 0.123 |  | 6.31 | 0.097 | 52.50% |  |  |
| homozygote model | Overall | 8 |  | 0.565 (0.383,0.834) | 0.004 |  | 16.61 | 0.020 | 57.90% |  | Random |
|  | OA | 1 |  | 0.952 (0.447,2.205) | 0.839 |  | 0.00 | - | 0.00% |  |  |
|  | IVDD | 3 |  | 0.344 (0.226,0.525) | 0.000 |  | 0.35 | 0.838 | 0.00% |  |  |
|  | RA | 4 |  | 0.704 (0.443,1.119) | 0.138 |  | 5.61 | 0.132 | 46.60% |  |  |
| heterozygote model | Overall | 8 |  | 0.746 (0.591,0.940) | 0.013 |  | 13.04 | 0.071 | 46.30% |  | Fixed |
|  | OA | 1 |  | 1.332 (0.652,2.721) | 0.431 |  | 0.00 | - | 0.00% |  |  |
|  | IVDD | 3 |  | 0.442 (0.288,0.679) | 0.000 |  | 0.86 | 0.649 | 0.00% |  |  |
|  | RA | 4 |  | 0.897 (0.660,1.219) | 0.489 |  | 2.52 | 0.472 | 0.00% |  |  |
| dominant model | Overall | 8 |  | 0.656 (0.461,0.934) | 0.019 |  | 16.06 | 0.025 | 56.40% |  | Random |
|  | OA | 1 |  | 1.165 (0.592,2.289) | 0.659 |  | 0.00 | - | 0.00% |  |  |
|  | IVDD | 3 |  | 0.382 (0.253,0.577) | 0.001 |  | 0.56 | 0.755 | 0.00% |  |  |
|  | RA | 4 |  | 0.820 (0.591,1.137) | 0.234 |  | 3.63 | 0.304 | 17.40% |  |  |
| recessive model | Overall | 8 |  | 0.794 (0.700,0.901) | 0.000 |  | 9.94 | 0.192 | 29.60% |  | Fixed |
|  | OA | 1 |  | 0.768 (0.450,1.310) | 0.332 |  | 0.00 | - | 0.00% |  |  |
|  | IVDD | 3 |  | 0.694 (0.576,0.837) | 0.001 |  | 0.21 | 0.900 | 0.00% |  |  |
|  | RA | 4 |  | 0.903 (0.754,1.082) | 0.269 |  | 5.76 | 0.124 | 47.90% |  |  |
| *Abbreviations: M, model used for meta-analysis; CI, confidence interval; ORs, odds ratios; N, number of studies included in each analysis.* | | | | | | | | | | | |
|  |  |  |  |  |  |  |  |  |  |  |  |
