# Supplementary material for: Association between Fas/FasL gene polymorphism and musculoskeletal degenerative diseases: a meta-analysis
Source: BMC Musculoskelet Disord. 2018 May 7;19:137. doi: 10.1186/s12891-018-2057-z (PMC5938814; doi:10.1186/s12891-018-2057-z)
Supplement: Supplementary file 4 — Table S4. Summary of meta-analysis for the association of FASL rs5030772 and rs763110 polymorphisms with musculoskeletal degenerative diseases leveled by race groups. (DOCX 22 kb) [file 12891_2018_2057_MOESM4_ESM.docx]

| **TABLE S4. Summary of meta-analysis for the association of FASL rs5030772 and rs763110 polymorphisms with musculoskeletal degenerative diseases leveled by race groups** | | | | | | | | | | | |
| --- | --- | --- | --- | --- | --- | --- | --- | --- | --- | --- | --- |
| **Genetic Model** | **Stratifcations** | **N** |  | **ORs ananlysis** | |  | **Heterogeneity Analysis** | | |  | **M** |
|  |  |  |  | **polled ORs (95% CI)** | **P value** |  | **χ2** | **P_heterogeneity_** | **I^2^ (%)** |  |  |
| FASL rs5030772 |  |  |  |  |  |  |  |  |  |  |  |
|  |  |  |  |  |  |  |  |  |  |  |  |
| allele model | Caucasoid | 3 |  | 0.863 (0.658,1.130) | 0.284 |  | 2.30 | 0.316 | 13.20% |  | Fixed |
| homozygote model | Caucasoid | 3 |  | 0.772 (0.397,1.502) | 0.446 |  | 1.40 | 0.496 | 0.00% |  | Fixed |
| heterozygote model | Caucasoid | 3 |  | 0.875 (0.622,1.232) | 0.445 |  | 0.93 | 0.629 | 0.00% |  | Fixed |
| dominant model | Caucasoid | 3 |  | 0.856 (0.621,1.180) | 0.344 |  | 1.62 | 0.445 | 0.00% |  | Fixed |
| recessive mode | Caucasoid | 3 |  | 0.800 (0.414,1.546) | 0.507 |  | 1.10 | 0.576 | 0.00% |  | Fixed |
|  |  |  |  |  |  |  |  |  |  |  |  |
| FASL rs763110 |  |  |  |  |  |  |  |  |  |  |  |
|  |  |  |  |  |  |  |  |  |  |  |  |
| allele model | Overall | 8 |  | 0.780 (0.671,0.907) | 0.001 |  | 14.31 | 0.046 | 51.10% |  | Random |
|  | Caucasoid | 3 |  | 0.777 (0.626,0.964) | 0.022 |  | 1.83 | 0.401 | 0.00% |  |  |
|  | Chinese | 4 |  | 0.772 (0.603,0.989) | 0.041 |  | 12.31 | 0.006 | 75.60% |  |  |
|  | Others | 1 |  | 0.795 (0.535,1.182) | 0.258 |  | 0 | - | - |  |  |
| homozygote model | Overall | 8 |  | 0.565 (0.383,0.834) | 0.004 |  | 16.61 | 0.020 | 57.90% |  | Random |
|  | Caucasoid | 3 |  | 0.624 (0.367,1.061) | 0.082 |  | 2.75 | 0.253 | 27.20% |  |  |
|  | Chinese | 4 |  | 0.492 (0.239,1.013) | 0.054 |  | 13.71 | 0.003 | 78.10% |  |  |
|  | Others | 1 |  | 0.674 (0.324,1.402) | 0.291 |  | 0 | - | - |  |  |
| heterozygote model | Overall | 8 |  | 0.737 (0.527,1.030) | 0.074 |  | 13.04 | 0.071 | 46.30% |  | Random |
|  | Caucasoid | 3 |  | 0.947 (0.582,1.542) | 0.827 |  | 2.73 | 0.255 | 26.80% |  |  |
|  | Chinese | 4 |  | 0.601 (0.336,1.073) | 0.085 |  | 8.5 | 0.037 | 64.70% |  |  |
|  | Others | 1 |  | 0.742 (0.370,1.487) | 0.4 |  | 0 | - | - |  |  |
| dominant model | Overall | 8 |  | 0.656 (0.461,0.934) | 0.019 |  | 16.06 | 0.025 | 56.40% |  | Random |
|  | Caucasoid | 3 |  | 0.806 (0.499,1.303) | 0.38 |  | 2.94 | 0.23 | 32.00% |  |  |
|  | Chinese | 4 |  | 0.533 (0.275,1.034) | 0.063 |  | 11.96 | 0.008 | 74.90% |  |  |
|  | Others | 1 |  | 0.711 (0.378,1.337) | 0.29 |  | 0 | - | - |  |  |
| recessive model | Overall | 8 |  | 0.763 (0.647,0.900) | 0.001 |  | 9.94 | 0.192 | 29.60% |  | Random |
|  | Caucasoid | 3 |  | 0.647 (0.465,0.901) | 0.01 |  | 0.69 | 0.709 | 0.00% |  |  |
|  | Chinese | 4 |  | 0.786 (0.618,0.998) | 0.048 |  | 7.53 | 0.057 | 60.10% |  |  |
|  | Others | 1 |  | 0.807 (0.443,1.469) | 0.482 |  | 0 | - | - |  |  |
| *Abbreviations: M, model used for meta-analysis; CI, confidence interval; ORs, odds ratios; N, number of studies included in each analysis.* | | | | | | | | | | | |
|  |  |  |  |  |  |  |  |  |  |  |  |
